# The F-bZIP-regulated Zn deficiency response in land plants

**DOI:** 10.1007/s00425-022-04019-6

**Published:** 2022-11-08

**Authors:** Ana G. L. Assunção

**Affiliations:** 1 Department of Plant and Environmental Sciences, University of Copenhagen, 1871 Frederiksberg, Denmark; 2 CIBIO-InBIO, Research Centre in Biodiversity and Genetic Resources, University of Porto, 4485-661 Vairão, Portugal

**Keywords:** Zinc deficiency, Micronutrient homeostasis, F-bZIP transcription factors, Zn sensor, Transport, Crop biofortification

## Abstract

**Main conclusion:**

This review describes zinc sensing and transcriptional regulation of the zinc deficiency response in Arabidopsis, and discusses how their evolutionary conservation in land plants facilitates translational approaches for improving the Zn nutritional value of crop species.

**Abstract:**

Zinc is an essential micronutrient for all living organisms due to its presence in a large number of proteins, as a structural or catalytic cofactor. In plants, zinc homeostasis mechanisms comprise uptake from soil, transport and distribution throughout the plant to provide adequate cellular zinc availability. Here, I discuss the transcriptional regulation of the response to zinc deficiency and the zinc sensing mechanisms in Arabidopsis, and their evolutionary conservation in land plants. The Arabidopsis F-group basic region leucine-zipper (F-bZIP) transcription factors bZIP19 and bZIP23 function simultaneously as sensors of intracellular zinc status, by direct binding of zinc ions, and as the central regulators of the zinc deficiency response, with their target genes including zinc transporters from the ZRT/IRT-like Protein (ZIP) family and nicotianamine synthase enzymes that produce the zinc ligand nicotianamine. I note that this relatively simple mechanism of zinc sensing and regulation, together with the evolutionary conservation of F-bZIP transcription factors across land plants, offer important research opportunities. One of them is to use the F-bZIP-regulated zinc deficiency response as a tractable module for evolutionary and comparative functional studies. Another research opportunity is translational research in crop plants, modulating F-bZIP activity as a molecular switch to enhance zinc accumulation. This should become a useful plant-based solution to alleviate effects of zinc deficiency in soils, which impact crop production and crop zinc content, with consequences for human nutrition globally.

## Introduction—Zn in biological systems

The micronutrient zinc (Zn) is essential for all living organisms because of its presence in a large number of proteins, as a structural or catalytic cofactor (Coleman 1992). It is estimated that Zn metalloproteins, i.e. proteins that require Zn ions for their physiological functions, represent nearly 10% of the proteome in eukaryotes (Andreini et al. 2006, 2009). Catalytic Zn sites are present in all six classes of enzymes, including a variety of key metabolic enzymes such as dehydrogenases, carbonic anhydrase, alkaline phosphatase, Cu/Zn superoxide dismutase (SOD) and RNA polymerase (Vallee and Auld 1990)*.* Many proteins have structural Zn sites, and Zn^2+^ binding to these sites is critical for stabilizing the tertiary structures of proteins that often mediate interactions with other proteins, nucleic acids or lipids. The Zn-finger structure is one of the most abundant, and mediates direct interactions of transcription factors with DNA. Zn-finger proteins are involved in the regulation of several cellular processes (Colvin et al. 2010). Zn is present in the divalent cation state (Zn^2+^) in biological systems, and it is the second most abundant metal micronutrient in organisms, after iron (Fe) (Broadley et al. 2007). Zn lacks biological redox activity, as a result of filled d-shell orbitals, and rather functions as a Lewis acid able to accept a pair of electrons (Colvin et al. 2010)*.* The abundance of Zn metalloproteins relates with the relatively strong affinity of Zn to various protein and peptide ligands. Zn interacts predominantly with nitrogen, sulphur, and oxygen donors from the side chains of the amino acids histidine, cysteine, and glutamate/aspartate, respectively, establishing most often tetrahedral coordination geometries*.* Because it is characterized by a fast ligand exchange and flexibility to accommodate multiple coordination geometries, Zn is a versatile protein cofactor (Vallee and Falchuk 1993; Krężel and Maret 2016). Biological redox processes, such as photosynthesis, mitochondrial respiration and nitrogen fixation, rely on the redox-active metal micronutrients Fe, manganese (Mn), cupper (Cu) and molybdenum (Mo), respectively, as cofactors in protein complexes involved in electron transfer reactions. Uncontrolled redox reactions can cause an imbalanced production of reactive oxygen species (ROS) and oxidative stress. In contrast to this, the lack of redox activity of Zn makes it a stable and safer ion in the proximity of sensitive macromolecules, in particular DNA in the nucleus (Maret and Li 2009; Sinclair and Krämer 2012). Nonetheless, Cu, Zn, Fe and Mn are also cofactors of SOD enzymes, which are associated with ROS scavenging.

Plants take up Zn^2+^ predominantly as a free ion from the soil, according to current knowledge. Zn available for plant acquisition is in water-soluble and exchangeable (adsorbed to soil particles) fractions, whereas most Zn in the soil is in the insoluble and mineral fraction (> 90%). Plant-available Zn depends on various soil characteristics (soil type, pH, organic matter, biota) but also on plant-mediated acidification and secretion of organic compounds, which influence Zn solubilisation (Broadley et al. 2007). Deficiency of plant-available Zn in soils is common and widespread globally, for example in calcareous or sandy soils, or highly weathered and leached tropical soils. As a result, Zn deficiency is the most commonly encountered and widespread micronutrient deficiency affecting crops (Broadley et al. 2007; Alloway 2008). Most crop plants require Zn concentrations in leaves between 15–30 and 100 mg Zn kg^−1^ DW for optimal growth. Below this concentration, symptoms of deficiency can become visible, including leaf chlorosis as well as reduced growth, negatively affecting yield and grain Zn content (Marschner 1995; White and Broadley 2011). Since there are many Zn metalloproteins, deficiency of Zn affects several critical biochemical processes, including the metabolism and scavenging of ROS following impaired Cu/Zn SOD activity (Sinclair and Krämer 2012)*.* Zn deficient soils and Zn deficient crops have a global impact on human nutrition, with one-third of the world’s human population at high risk of Zn malnutrition, in particular in developing regions with cereal crop-based diets from Zn-deficient soils (Wessells and Brown 2012). Considering a foreseeable increase in plant-based diets, micronutrient deficiencies, including Zn deficiency, might also become a concern in more developed regions (Assunção et al. 2022). In humans, Zn deficiency increases infant mortality and has a negative effect on growth, cognitive development and the immune system, with Zn being important for the functionality of the immune response (Prasad 2013). Therefore, strategies to improve the Zn nutritional value of crops through biofortification, and also increasing awareness for the importance of micronutrients in crop production and quality, can contribute to tackling the global Zn deficiency problem (Bouis and Saltzman 2017; Assunção et al. 2022).

To maintain the concentration of Zn within physiological limits, organisms rely on a tightly regulated network of homeostasis mechanisms that responds to external supply and internal requirements. In plants, these mechanisms include the uptake of Zn from soil, its transport and distribution throughout the plant, providing sufficient Zn to all cells, at different developmental stages and environmental conditions. Regulated Zn transport across membranes by Zn transporters, in combination with Zn ligands, play key roles in Zn homeostasis (Sinclair and Krämer 2012). Low-molecular-weight (LMW) compounds that form complexes with metals contribute to intercellular symplastic mobility and long-distance distribution, and mediate vacuolar storage under conditions of metal excess (Clemens 2019). The LMW ligand nicotianamine (NA), a non-proteinogenic amino acid, is a Zn ligand, and other LMW compounds implicated in plant Zn homeostasis are candidate Zn ligands, such as the glutathione-derived small peptide phytochelatin (PC), histidine and organic acids (citrate and malate) (Verbruggen et al. 2009; Clemens et al. 2013). The plant membrane transporters of Zn include members from major families of divalent metal transporters. These are the ZIP (ZRT/IRT-like Protein), CDF/MTP (Cation Diffusion Facilitator, also known as Metal Tolerance Proteins) and HMA (P_1B_-type Heavy Metal ATPases), which are ubiquitous in all kingdoms of life (Guerinot 2000; Hussain et al. 2004; Krämer 2005). The Zn transporter members from these families function in opposite directions to maintain cellular Zn homeostasis; i.e., they either mediate Zn influx into cytosol (ZIP), Zn efflux into organelles (CDF/MTP), or use ATP hydrolysis to pump Zn out of the cytosol into the apoplast or into organelles (HMA). These and other transporter families that are involved in plant Zn homeostasis are described in previous reviews (Haydon and Cobbett 2007; Curie et al. 2009; Palmgren and Nissen 2011; Sinclair and Krämer 2012). In addition to the cytosol and the nucleus, but still poorly characterized in plants, Zn-requiring proteins are possibly present in all major organelles including endoplasmic reticulum, Golgi, mitochondria, chloroplast and vacuole. The latter is the major site for storage of excess Zn and also a source for Zn remobilisation in periods of deficiency (Eide 2020; Clemens 2021)*.* To prevent unspecific interactions and off-target binding to proteins, the presence of intracellular free Zn ions is tightly controlled. In eukaryotic cells, the total Zn concentration under steady-state conditions is estimated in the high µM range, whereas the fraction of Zn that is free or loosely bound to easily replaceable ligands and not tightly bound to proteins, termed “free” or “labile” Zn, is estimated in the pM-nM range. Compared to the pool of bound Zn, the “free” Zn pool is thus several orders of magnitude smaller, and it represents the available supply of Zn to nascent proteins (Colvin et al. 2010; Eide 2020; Clemens 2021). In plants, the use of genetically encoded Zn-responsive fluorophores in Arabidopsis root cells estimated the “free” Zn concentrations to around 400 pM, and total Zn concentration around 100 µM (Lanquar et al. 2013). The LMW ligands also have a function in buffering the cytosolic “free” Zn pool, though the specific contribution of each ligand compound is not known, mainly due to experimental difficulties (Clemens 2019). Thus, Zn transporters localized to the plasma membrane and various subcellular organelles, in combination with Zn ligands, are involved in Zn acquisition, intra- and inter-cellular Zn mobility, storage and distribution at the plant level, and are key players in the network that maintains cellular and systemic Zn homeostasis*.*

When exposed to limiting Zn availability, plants and other organisms respond with mechanisms to acclimate to Zn deficiency and maintain Zn homeostasis. Among eukaryotes, these mechanisms are best studied in the yeast model organism *Saccharomyces cerevisiae*. Here the Zap1 transcription factor is the central regulator of the Zn deficiency response (Zhao and Eide 1997), and the Zap1 regulon informs on how cells respond to Zn deficiency stress (Eide 2020). The mechanisms include, primarily, the regulation at the transcriptional level of transporters and other components of Zn homeostasis (Choi and Bird 2014). Additionally, other strategies, named as adaptive responses, were identified in yeast, such as altering metabolic processes to spare limiting Zn, prioritizing Zn usage, or increasing tolerance to oxidative stress under severe Zn deficiency (Wang et al. 2018; Eide 2020). Next, I review the current knowledge on the transcriptional regulation of the Zn deficiency response and the Zn sensing mechanisms in plants.

## Transcriptional regulation of the Zn deficiency response in Arabidopsis

### bZIP19 and bZIP23 transcription factors

In the model plant *Arabidopsis thaliana* (Arabidopsis), the bZIP19 and bZIP23 transcription factors are the central regulators of the Zn deficiency response. They transcriptionally activate the expression of genes involved in Zn homeostasis and in the response to Zn deficiency (Assunção et al. 2010; Castro et al. 2018). bZIP19 and bZIP23 are members of the basic region leucine-zipper (bZIP) family of eukaryotic transcription factors. These are characterized by a highly conserved bZIP domain comprising a basic region, involved in specific DNA binding, and a leucine-zipper for protein–protein interaction and dimerization. To be targeted to the nucleus, bZIPs carry a nuclear localization signal that is located within the basic region. Dimerization properties and DNA-binding site specificity are critical for the activity of bZIP transcription factors, which bind the DNA as dimers (Vinson et al. 2006; Amoutzias et al. 2006). In Arabidopsis, bZIPs are involved in different processes including development, metabolism, signalling and stress responses. The Arabidopsis bZIP family was subdivided according to sequence similarities and functional features, and a revised classification identifies 78 members classified into 13 groups (named A-M) (Jakoby et al. 2002; Dröge-Laser et al. 2018). bZIP19, bZIP23 and a third member, bZIP24, form the F group bZIP transcription factors (F-bZIP), which are characterized by a motif located at the N terminus. This F-bZIP signature motif comprises two short regions rich in cysteine (Cys) and histidine (His) amino acids (Assunção et al. 2013). These three Arabidopsis F-bZIP transcription factors are nuclear-localized and functional as homodimers (Yang et al. 2009; Inaba et al. 2015; Lilay et al. 2019). bZIP19 and bZIP23 share 69% amino acid sequence identity, and 28% and 32% with bZIP24, respectively. bZIP24 is reported as a regulator of the salt stress tolerance (Yang et al. 2009) and does not play a major role in the Zn deficiency response (Lilay et al. 2019).

bZIP19 and bZIP23 are required for the Zn deficiency response. Contrary to Arabidopsis wild-type plants, the *bzip19 bzip23* double mutant (*bzip19/23*) fails to cope with the stress of Zn deficiency and develops a Zn deficiency hypersensitive phenotype. The mutant is functionally complemented by constitutive expression of either bZIP19 or bZIP23 (Assunção et al. 2010). Analysis of the single mutants shows no visible phenotype in *bzip23*, and a milder sensitivity to Zn deficiency in *bzip19* in comparison with *bzip19/23*, indicating that bZIP19/23 are partially redundant (Assunção et al. 2010; Inaba et al. 2015; Nazri et al. 2017). Partial, as opposed to complete, redundancy is possibly explained by the non-overlapping expression of *bZIP19* and *bZIP23*, as observed in promoter-GUS fusion analyses that show *bZIP19* mostly expressed in roots and *bZIP23* in shoots (Lilay et al. 2019). This pattern of expression for *bZIP19* and *bZIP23* appears to agree with the expression of their target genes in the root of *bzip19* and *bzip23* single mutants in response to Zn deficiency: the expression pattern in *bzip19* is similar to that in the double mutant *bzip19/23*, whereas the expression pattern in *bzip23,* likely with endogenous bZIP19 in the root*,* is similar to the wild-type (Inaba et al. 2015). Further analysis, including more detailed tissue-specific gene expression, and other regulatory levels as dimerization (homodimer vs heterodimer activity), should provide more knowledge on the individual roles of bZIP19 and bZIP23.

In plants exposed to Zn deficiency, bZIP19 and bZIP23 activate the expression of target genes by binding to the Zinc Deficiency Response Element (ZDRE) in their promoters (Assunção et al. 2010). The ZDRE is a 10-bp imperfect palindromic sequence, with consensus (RTGTCGACAY) and its discovery represented a novel plant bZIP-DNA binding sequence. Generally, plant bZIP transcription factors preferentially bind to hexamer DNA sequences with an ACGT-core, as found in the A-, C- or G-box (T/G/CACGTG/C/A), but there are some examples of nonpalindromic binding sites that do not contain the ACGT-core sequence (Jakoby et al. 2002). The specificity of the DNA recognition results from the cis-element sequence combined with the bZIP domain basic region that contacts the DNA. The latter is highly conserved, thus binding preferences must be determined by only subtle differences in its sequence (Llorca et al. 2014). The binding of bZIP19 and bZIP23 to ZDREs was observed in a yeast-one-hybrid screen that identified both proteins, and subsequently confirmed by a pull-down in vitro assay (Assunção et al. 2010). Transcriptomic profiling comparing Arabidopsis *bzip19/23* and wild-type roots and shoots, grown under Zn deficiency, revealed only a very small number (< 20) of differentially expressed genes. This number decreased to less than half when comparing *bzip19/23* and wild-type grown at sufficient or excess Zn supply, suggesting that the role of bZIP19 and bZIP23 is specific to the Zn deficiency response. These differentially expressed genes were mostly induced under Zn deficiency, and include Zn homeostasis-related genes, i.e. encoding members of the ZIP transporter family and nicotianamine synthase (NAS) enzymes that produce the Zn ligand NA (Castro et al. 2017). ZIP proteins were also identified in proteomic profiling comparing *bzip19* and wild-type roots under Zn deficiency (Inaba et al. 2015). This collection of bZIP19/23-regulated genes provides valuable insight on how plants respond to Zn deficiency.

### bZIP19/23-regulated *ZIP* and *NAS* genes

As noted earlier, the bZIP19/23-regulated *ZIP* genes are members of the ZIP family of divalent cation transporters. The ZIP transporters, which were first identified in plants (Eide et al. 1996), are transporters of Zn, Fe and Mn, and mediate uptake into the cytosol, either from the extracellular space or from organelles. They are also involved in the uptake of toxic metal Cd (Guerinot 2000; Hu 2021). In Arabidopsis, within the 15 ZIP members, eight are regulated by bZIP19 and bZIP23 (*AtZIP1/3/4/5/9/10/12* and *AtIRT3*). They have ZDREs in the promoter, their expression is induced by Zn deficiency and is suppressed in the *bzip19/23* mutant, where it can be restored upon functional complementation with bZIP19 or bZIP23 (Assunção et al. 2010; Lilay et al. 2019). Except for ZIP5, these ZIP members were all shown to be able to mediate cellular Zn uptake by heterologous yeast complementation (Grotz et al. 1998; Lin et al. 2009; Assunção et al. 2010; Milner et al. 2013; Lee et al. 2021). The *ZIP4* promoter, containing two ZDREs, was successfully used as bait in the yeast-one-hybrid screening leading to the identification of bZIP19 and bZIP23 (Assunção et al. 2010). Furthermore, the *ZIP4* promoter fused with GUS (*pZIP4::GUS*) in the *bzip19/23* mutant background indicated that *ZIP4* is only regulated by bZIP19/23 (Castro et al. 2017). This allowed using the *pZIP4::GUS* as a Zn deficiency marker, and as an in planta reporter system to obtain functional insight into the regulatory mechanism of bZIP19 and bZIP23 transcription factors. The physiological function of individual ZIP transporters in plant micronutrient homeostasis is generally not well elucidated, which might be a consequence of their possible functional redundancy (Sinclair and Krämer 2012; Lee et al. 2021). More information on tissue and subcellular localizations and transport properties of ZIP transporters, such as substrate specificity and affinity, will contribute to clarify the function of each of the bZIP19/23-regulated ZIPs in the Arabidopsis Zn deficiency response. In the yeast *S. cerevisiae*, the expression of the genes encoding ZIP family Zn transporters Zrt1 and Zrt2 is activated by the Zap1 transcription factor. Both transporters are localized at the plasma membrane, but Zrt1 is involved in high-affinity Zn uptake while Zrt2 is involved in low-affinity Zn uptake (Zhao and Eide 1997). The high affinity transporters work in tandem with low affinity transporters to control the transition from replete to limiting Zn supply conditions, with Zrt1 playing a major role under Zn limiting conditions (Eide 2020). It is therefore likely that some bZIP19/23-regulated *ZIP* genes encode high-affinity Zn transporters to ensure Zn uptake and acquisition under limiting Zn availability.

The small set of bZIP19/23-regulated genes includes two Arabidopsis *NAS* (*AtNAS2/4*). Arabidopsis has in total four *NAS* genes, which encode the enzyme producing the LMW ligand NA (Bauer et al. 2004). NA is a mobile compound able to form complexes with Zn, Fe, Mn, Cu, Co and Ni (Curie et al. 2009). NA contributes to Zn and Fe mobilization, with a role in Zn radial movement in roots and long-distance Zn translocation, facilitating xylem and phloem Zn movements (Clemens 2019), and with a role in Fe movement and accumulation in seeds (Schuler et al. 2012). Like the bZIP19/23-regulated *ZIP* genes, *NAS2* and *NAS4* contain ZDREs in the promoter and their expression is transcriptionally activated by bZIP19/23 in response to Zn deficiency (Assunção et al. 2010)*.* The regulation of *NAS* genes seems to be complex and controlled by multiple mechanisms. In Arabidopsis, bHLH and MYB transcription factors that are part of the Fe deficiency response transcriptional network, also regulate the expression of *NAS* genes, and possibly have a balancing role in cross-talk between Fe and Zn homeostasis (Palmer et al. 2013; Chen et al. 2018).

Zn homeostasis is a highly controlled process, and other players and levels of regulation will likely be involved. For example, analysis of Zn-deficient Arabidopsis upon Zn re-supply revealed novel dynamics of proteome, transcripts and ionome that suggest Zn homeostasis candidates not evident under steady-state conditions (Arsova et al. 2019). Post-translational regulatory mechanisms have been described in ZIP family transporters. For example, the Zn uptake transporters Zrt1 from *S. cerevisiae* and the mammalian Zip4 are ubiquitinated, sorted for endocytosis and degraded to prevent overaccumulation of Zn (Bird 2015; Hu 2021). Another example of post-translational regulation of a ZIP member comes from the Arabidopsis IRT1 transporter, which has a major role in Fe uptake and it is transcriptionally upregulated in roots by Fe deficiency (Vert et al. 2002). The IRT1 transporter is post-translationally regulated by non-Fe metals (Zn, Mn and Co), which are also transported by IRT1, but it is their direct binding to a cytosolic His-rich stretch in IRT1 that triggers IRT1 ubiquitination, endocytosis and degradation. This metal sensing mechanism in IRT1 prevents excess uptake of non-Fe metals when Fe is required (Dubeaux et al. 2018). Also important may be the integration of the bZIP19/23-regulated Zn deficiency response with other micro- and macronutrient homeostasis networks. The Arabidopsis lysophosphatidylcholine acyltransferase (LPCAT1) was identified as a key determinant of phosphate (Pi) accumulation in shoots under Zn deficiency (Kisko et al. 2018). In the identified pathway, bZIP23, and not bZIP19, binds to a novel motif sequence present in the *LPCAT1* 5´UTR and this is suggested to block *LPCAT1* transcription at Zn deficiency, which in turn leads to increased expression of Pi transporter gene *PHT1;1* and increased shoot Pi accumulation. *LPCAT1* expression is induced under Zn sufficiency, in comparison to Zn deficiency, and it is also induced under Zn deficiency in the *bzip23* mutant background (Kisko et al. 2018). The transcriptional repression by bZIP23 contrasts with the bZIP19/23 transcriptional activation of target genes upon binding to the ZDRE at Zn deficiency, as reviewed here. This could be related with the bZIP23-specific binding to the novel motif sequence and its characteristics, but further research is necessary to elucidate the mechanism. In addition to interactions with Pi homeostasis, possible interactions between the bZIP19/23-regulatory network and Fe, Mn and Cu homeostasis were suggested based on ionomic profiling comparing Arabidopsis *bzip19/23* and wild-type plants (Lilay et al. 2019). Metal substrates other than Zn in the ZIP transporters encoded by the *ZIP* target genes would support that putative crosstalk. Known interactions between mineral nutrients, as Pi, Zn and Fe (Thiébaut and Hanikenne 2021), support the concept that ionomic networks in plants are coordinately regulated and need to be viewed as a whole (Huang and Salt 2016).

## Zn sensors and sensing Zn deficiency in Arabidopsis

### bZIP19 and bZIP23 function as Zn sensors of intracellular Zn status

**Fig. 1:**
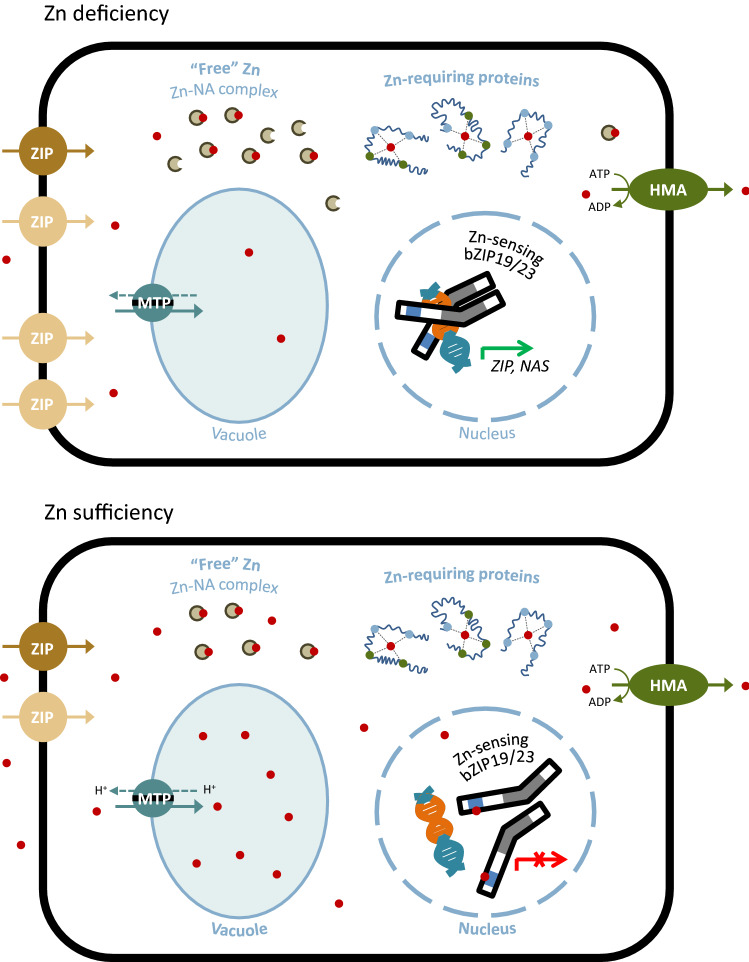
Simplified scheme of cellular response to Zn deficiency and Zn sensing. Under Zn deficiency, the bZIP19 and bZIP23 transcription factors, localized in the nucleus, bind to the Zn Deficiency Response Elements (ZDRE) in the promoter of their target genes and activate transcription, which include several *ZIP* and *NAS* genes. The relative numbers of ZIP transporters and nicotianamine (NA) symbols in the figure are extrapolated from transcript abundance of *ZIP* and *NAS* genes. The ZIP transporters mediate Zn uptake into the cytosol, and are likely involved in cellular Zn uptake in plants. They are depicted here as high- and low-affinity Zn transporters (light brown and dark brown symbols, respectively). It is suggested that some of the bZIP19/23-regulated *ZIP* genes should encode high-affinity Zn transporters to ensure cellular Zn uptake and acquisition under limiting Zn availability (as discussed in “Transcriptional regulation of the Zn deficiency response in Arabidopsis”). The Zn^2+^ ions (red dots) inside the cell are free or loosely bound to easily replaceable ligands, corresponding to the “free” or “labile” Zn pool. The Zn-requiring proteins are supplied with Zn from the “free” Zn pool, which becomes tightly bound to proteins (Colvin et al. 2010; Clemens 2021). NA contributes to inter-cellular and long-distance Zn distribution (Clemens 2019). In this simplified scheme, only NA is represented as a Zn-ligand (NA molecules and Zn–NA complexes) and it is suggested to also contribute to the cytosolic Zn buffer capacity at Zn deficiency (as discussed in “Zn sensors and sensing Zn deficiency in Arabidopsis”). Under Zn sufficiency, cellular free or loosely bound Zn ions from the “free” Zn pool, bind to the Cys/His-rich Zn Sensor Motif (ZSM) of bZIP19 and bZIP23 transcription factor proteins. The Zn-binding to the proteins affects their activity and halts target gene transcriptional activation (Lilay et al. 2021). An MTP transporter, a vacuolar membrane Zn^2+^⁄H^+^ antiporter, effluxes Zn into the vacuole, and an HMA Zn/Cd transporter P_1B_-type ATPase pumps Zn to the apoplast, for root xylem loading. Additional transporter localizations and other transporter families, not represented here, also contribute to Zn homeostasis (Sinclair and Krämer 2012; Clemens 2021)

The Arabidopsis bZIP19 and bZIP23 transcription factors function not only as the central regulators of the Zn deficiency response, but also as sensors of intracellular Zn concentration (Fig. 1). As noted earlier, the Cys and His amino acids from the F-bZIP signature motif are the predominant Zn-coordinating amino acids in Zn metalloproteins and this was the basis for a theoretical model proposing that Arabidopsis bZIP19 and bZIP23 can sense cellular Zn status via a direct binding of Zn ions to the Cys/His-rich motif (Assunção et al. 2013). Contrary to the yeast Zap1 transcription factor, the levels of which are transcriptionally autoregulated in response to Zn (Eide 2020), the transcript levels of *bZIP19* and *bZIP23* do not respond significantly to Zn supply indicating that bZIP19/23 Zn-dependent activity is not regulated at the transcriptional level (Inaba et al. 2015; Arsova et al. 2019; Lilay et al. 2019). Instead, the analysis of *ZIP* and *NAS* target gene expression, in Arabidopsis *bzip19/23* functionally complemented with *bZIP19* or *bZIP23*, suggested that Zn modulates bZIP19/23 activity at the protein level, and cellular Zn sufficiency represses their activity (Lilay et al. 2019). The hypothesis of bZIP19 and bZIP23 acting as Zn sensors was tested with an in vitro Zn-protein binding analysis, using size-exclusion chromatography-inductively coupled plasma mass spectrometry. The analysis, testing native protein and a protein variant with the Cys/His-rich motif deleted, showed that bZIP19 and bZIP23 bind to Zn ions in vitro and that the motif is required (Lilay et al. 2021). The biological relevance of this result was verified in planta using the *bzip19/23-pZIP4::GUS* reporter system to obtain insight on the activity of bZIP19/23. The absence of reporter activity in the *bzip19/23* mutant is restored upon complementation with the overexpression of bZIP19, showing a Zn-deficiency induced expression that decreases with increasing Zn supply, which is in line with the *ZIP4* Zn-responsive expression pattern (Castro et al. 2017; Lilay et al. 2021). When the reporter system is complemented with bZIP19 variants that harbour deletions or amino acid substitutions in the Cys/His-rich motif, then the reporter activity shows constitutive expression irrespective of the cellular Zn status. This indicated, on the one hand, that the motif-variants of bZIP19 are functional and activating transcription at the *ZIP4* promoter, but, on the other hand, that they are no longer responsive to changes in the plant cell’s physiological Zn status. These Zn-insensitive bZIP19 variants transcriptionally activate *ZIP* and *NAS* target genes constitutively (Lilay et al. 2021). Together, this is robust evidence that bZIP19 and bZIP23 act as Zn sensors through direct binding of Zn ions to their Cys/His-rich motif, now referred to as Zn-sensor motif (ZSM).

### Perspectives on the Zn binding structure and properties of the bZIP19 and bZIP23 sensors

**Fig. 2:**
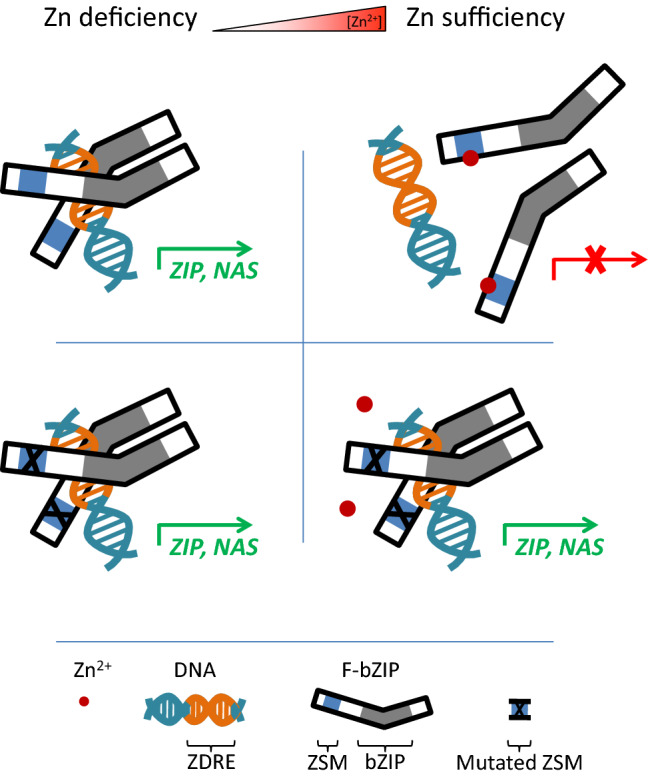
Scheme for the effect of Zn Sensor Motif (ZSM) variants in the F-bZIP function as Zn sensors of intracellular Zn status. The Arabidopsis bZIP19 and bZIP23 transcription factors also function as sensors of intracellular Zn concentration (Fig. 1). In the current working model, Zn binding to the ZSM elicits a protein conformational change that affects the activity of the bZIP19 and bZIP23 transcription factors. Since bZIP transcription factors function as dimers to bind to a specific DNA site (Vinson et al. 2006), it is plausible that a protein conformational change interferes with bZIP protein dimerization and DNA-binding or, alternatively, with protein stability or protein interactions (Assunção et al. 2013; Lilay et al. 2021). Mutated ZSM refers to full or partial (each of the two regions) deletions of the ZSM, or to amino acid substitutions of Cys and His residues by alanine as described by Lilay et al. (2021)

In vitro and in planta analyses revealed that Zn binding to the ZSM of bZIP19/23 is necessary to halt the transcriptional activation of their target genes (Lilay et al. 2021). Possibly Zn binding elicits a conformational change that affects the activity of the transcription factor (Fig. 2). One such mechanism was shown for the bacterial Zn uptake regulator (Zur) proteins that play an important role in maintaining Zn homeostasis by transcriptionally controlling Zn import and export (Mikhaylina et al. 2018). These Zur regulators act as Zn sensors, with Cys and His residues involved, and crystal structures together with other analyses showed that Zn binding drives a conformational change that is essential for activating the transcription factor and for DNA recognition (Liu et al. 2021). Considering that bZIP transcription factors function as dimers to bind to a specific DNA site (Rodríguez-Martínez et al. 2017), it is plausible that Zn binding to the ZSM has an allosteric effect in the bZIP19/23 protein structure that will affect its dimerization and DNA binding. For example, interference with the position or exposure of the leucine zipper or basic region from the bZIP domains could disable DNA contact and transcriptional activity (Schütze et al. 2008). Alternatively, Zn binding could affect protein interactions or even protein stability. Yet another possibility could be an effect of Zn binding on the protein subcellular targeting, and thus a Zn-dependent change in nuclear versus cytosolic localization, as described in other Arabidopsis bZIP transcription factors (Llorca et al. 2014), but this does not seem to occur since the nuclear localization of bZIP19 and bZIP23 is independent of cellular Zn status (Lilay et al. 2019).

**Fig. 3:**
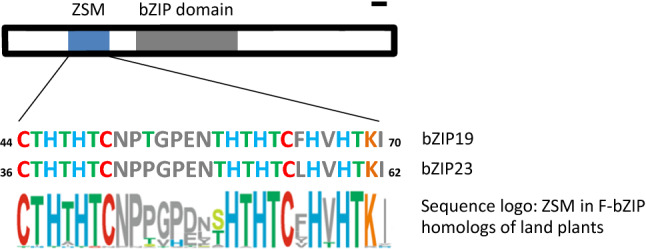
Schematic representation of the Zn Sensor Motif (ZSM) (blue) and bZIP domain (grey) of F-bZIP transcription factors. The amino acid sequence alignment of the Cys/His-rich ZSM in bZIP19 and bZIP23 are shown, comprising the two Cys/His-rich regions (Assunção et al. 2013). Cys (C), His (H), Thr (T) and Lys (K) are represented in red, blue, green and orange, respectively. Below the ZSM sequence alignment is a graphical representation of the ZSM sequence conservation in a comparison between F-bZIP homologs across land plants (Group 1 F-bZIPs from 24 species representing all major plant taxa) (Castro et al. 2017), where the height of letters indicates the degree of amino acid conservation in that position. The bZIP domain (grey) consists of a basic region for DNA-binding and the adjacent leucine zipper enabling bZIP dimerization (Dröge-Laser et al. 2018). The proportions of the F-bZIP protein scheme relates to the amino acid positions of Arabidopsis bZIP19 (scale bar on the top right represents 10 amino acids)

Constitutive and Zn-independent reporter activity was observed in all analysed bZIP19 ZSM variants, which include variants with full or partial (each of the two short regions) deletion of the motif, or with substitutions of all Cys and His amino acid residues, in different combinations, including a single Cys substitution (Lilay et al. 2021; Fig. 3). This indicates that the position and amino acid identity in the ZSM sequence, at least for all Cys and His residues, are essential for Zn binding to bZIP19/23 proteins. It suggests that only binding of Zn to the native ZSM elicits the required coordination geometry to trigger the sensing mechanism and halt the transcriptional activation activity. The bZIP19 ZSM variants with Cys or His substitutions still contain some of these amino acids in the ZSM, which suggests that the motif could still be able to bind Zn, but the Zn occupancy and coordination geometry does not cause the required effect (possibly a conformational change that triggers the sensing response). There are examples of bacterial metal sensors that do not adopt the required coordination geometry upon metal binding, due to binding of a different metal, and in spite of metal binding there is no triggering of the sensing mechanism (Blindauer 2015). The significance of the precise position and amino acid identity in the bZIP19/23 ZSM sequence is in agreement with the observed high level of evolutionary conservation of the ZSM sequence in F-bZIP homologs across land plants (Assunção et al. 2010; Castro et al. 2017). Graphical representation of the amino acid sequence conservation across species showed that the Cys and His residues and positions are very conserved in the motif. The analysis also found that several threonine (Thr) positions, one lysine (Lys), and the spacing between the two Cys/His-rich regions of the motif are conserved (Castro et al. 2017), suggesting that they are important, perhaps as structural elements supporting the exact Zn coordination geometry in the ZSM (Fig. 3). Information from phylogenetic analysis on the ZSM, in particular position and identity of conserved residues, in combination with the analysis of a range of bZIP19/23 ZSM variants and information from 3D protein structure analysis, will provide exciting insight on how Zn binds to bZIP19/23 and affects their regulatory activity*.*

In addition to protein structure and coordination with Zn, the Zn binding affinity and specificity of the bZIP19/23 sensors are important properties to investigate. The estimates of relative affinities for metal sensors and the estimates of buffered cytosolic metal concentrations show a remarkable correlation (Foster et al. 2014). As discussed earlier, the estimated “free” or “labile” Zn pool in cytosol is in the pM-nM range and considering that nuclear pores are permeable to free or small Zn-ligand complexes (Clemens 2021) this cytosolic Zn pool likely supplies the nucleus. Reported relative Zn affinity values of several bacterial Zn sensors are in the pM range (Blindauer 2015), and this will likely be the affinity range of bZIP19/23 sensors in line with the “free” Zn concentration range in Arabidopsis cells (Lanquar et al. 2013). The affinity of Zn sensors defines the detection threshold or upper limit for cytosolic free Zn^2+^ ion levels (Waldron et al. 2009; Foster et al. 2014)*.* Considering the role of bZIP19 and bZIP23 in the Zn deficiency response, their Zn affinity will set the upper limit of free Zn ion levels that defines sensing Zn deficiency or Zn sufficiency.

The abundance of Zn ligands can modulate the cytosolic buffering of “free” Zn in response to fluctuations in Zn supply (Clemens 2021) and, in response to Zn deficiency, the expression of the bZIP19/23-regulated *NAS* genes is activated. The Zn-ligand NA complex, in addition to the role in symplastic mobility and long-distance transport noted earlier, is also a candidate ligand in buffering the cytosolic “free” Zn pool in plants (Clemens 2021) (Fig. 1). The stability of metal-NA complexes is higher at pH values above 6.5, suggesting that NA is more likely a symplastic ligand, binding metals predominately within cells and the phloem (von Wirén et al. 1999). It is conceivable that an increase in cellular NA ligand in response to Zn deficiency modulates the cytosolic buffering of “free” Zn and decreases the level of free Zn ions to below the detection threshold of bZIP19/23 sensors, causing a delay in sensing Zn sufficiency. If so, the induced expression of *NAS2/4* genes in Arabidopsis in response to Zn deficiency could also have a role as a “proactive” mechanism to prevent a putative “Zn shock” upon induced expression of the *ZIP* Zn transporter genes. The “Zn shock” is described in yeast *S. cerevisiae*, where the vacuolar Zrc1 efflux transporter, a protein of the CDF family, undergoes transcriptional upregulation by the Zap1 transcription factor in Zn-limited cells. Zrc1 mediates Zn storage in vacuoles if excess Zn should suddenly accumulate in cells in which ZIP transporters Zrt1/2 are present at high levels (Eide 2009).

Regarding Zn sensor specificity, and discrimination from other metals, the activity of bZIP19 and bZIP23 seems to be specific to Zn deficiency. Other micronutrient deficiencies (Cu, Fe and Mn) did not trigger the *pZIP4::GUS* reporter system nor caused a phenotype in the *bzip19/23* mutant in relation to the wild-type. Instead, reporter activity only occurs upon Zn deficiency, despite the presence of sufficient Cu, Fe and Mn in the growth media, and it is only reduced upon Zn sufficiency (Nazri et al. 2017; Lilay et al. 2021). The stability of complexes between divalent metals and organic ligands follows the Irving–Williams series (Irving and Williams 1948), with the order Mn < Fe < Co < Ni < Cu > Zn, where Cu and Zn are more competitive over other metal nutrients. However, the metal requirements in vivo generally do not match the in vitro metal binding preferences, since correct metallation of a protein in the context of a cellular environment is governed by metal availability, metal homeostasis networks, and metal delivery pathways, among other factors (Foster et al. 2014). The delivery of Cu inside the cell to target proteins, transporters and organelles is carried out by Cu chaperone proteins (Burkhead et al. 2009). Analysis of chaperones that assist metal delivery to target enzymes identified chaperones for Cu, Fe, Ni, Co, but not for Zn (Foster et al. 2014). As discussed earlier, contrary to Cu and the less competitive Fe and Mn, Zn does not participate in redox reactions. In the nucleus there are many Zn-requiring proteins (Colvin et al. 2010). For example, in the human proteome, Zn is a cofactor for over 300 enzymes and is required for the function of over 2000 transcription factors (Jeong and Eide 2013). It is conceivable that the sensor specificity for Zn relies on the absence of other more reactive metals in the nucleus, which in a labile form would be able to compete with “free” Zn for the binding to the ZSM. Nonetheless, the binding of a different metal to the ZSM might not cause the required coordination geometry to trigger the sensing mechanism, as discussed above. Also, in conditions of extreme Zn deficiency or metal toxicity, with homeostasis possibly compromised, it can be speculated that the binding of Zn to bZIP19/23 ZSM would be affected by oxidative stress when the sulphur donor from Cys is preferentially oxidized, interfering with its ability to bind to Zn (Hübner and Haase 2021). Current knowledge indicates that the sensing by bZIP19/23 transcription factors is specific for Zn, likely relying on the remarkably precise plant metal homeostasis networks (Clemens 2001).

Transcription factors involved in the regulation of Fe and Cu deficiency responses in plants have also been identified (Kobayashi and Nishizawa 2012; Bernal et al. 2012). For Fe, there are complex transcriptional regulatory networks composed of various transcription factors (Vélez-Bermúdez and Schmidt 2022). These include several members of the basic helix-loop-helix (bHLH) family, and post-translational regulation can significantly affect the regulatory activities of the transcription factors (Gao and Dubos 2021; Wu and Ling 2019). For Cu, the plant-specific SQUAMOSA promoter binding protein (SBP)-domain transcription factors, SPL7 in Arabidopsis, and the close homolog CRR1 from the algae *Chlamydomonas reinhardtii*, are the major known transcription factors mediating Cu deficiency responses (Sommer et al. 2010; Bernal et al. 2012). SPL7 directly binds to Cu response elements (CuRE) located in the promoter of Cu responsive genes, and transcriptionally activates genes involved in Cu uptake and mobilization. SPL7 also mediates miRNA-dependent down-regulation of Cu metalloprotein transcripts, as a mechanism to spare Cu under conditions of deficiency (Yamasaki et al. 2009; Garcia-Molina et al. 2014). The SBP domain is a highly conserved DNA-binding domain with conserved Cys and His residues involved in Zn-binding and Zn-finger structures. The SBP domain from CRR1 was shown to bind Cu ions in vitro (Sommer et al. 2010), but, it is not fully understood how SPL7 and CRR1 are regulated by Cu. Thus, for the transcription factors that regulate Fe and Cu deficiency responses, the mechanisms underlying sensing of these micronutrients are not yet identified. Perhaps it can be speculated that the non-redox nature of Zn and its flexibility to accommodate multiple coordination geometries contribute to the identified dual role of bZIP19/23 as transcription factors and cellular Zn sensors, and that for other redox-active metal micronutrients more complex sensing mechanisms are required.

Mechanisms of sensing cytosolic metal status have also been reported in the context of post-translational regulation of metal transporters, and those also involve conserved Cys and/or His amino acids in the transporter proteins. One example is the IRT1 transporter that, as described earlier, has a cytosolic His-rich metal sensor involved in auto-regulating protein degradation (Dubeaux et al. 2018). The MTP1 Zn vacuolar transporter, from the CDF family, contains a Zn binding cytosolic His-rich loop suggested to have an auto-regulatory role in the transport activity through induced structural changes (Tanaka et al. 2013; Krämer 2005). And the HMA transporter pumps, of the P_1B_-type ATPase family, have N- and C-terminal cytosolic extensions that contain Cys- and His-rich domains, which may be involved in metal sensing and the regulation of transport activity (Laurent et al. 2016; Bækgaard et al. 2010).

Finally, the regulation of the Zn deficiency response at the organismal level involves not only the cellular Zn sensing, but also intercellular and inter-organ communication of Zn status through systemic signalling. The analysis of Arabidopsis *MTP2* and *HMA2* Zn transporter-encoding genes, of the CDF and P_1B_-ATPase families, respectively, showed an increase in their transcript levels in the root in response to physiological Zn deficiency in the shoot. This provided evidence of a shoot-borne signal and systemic regulation of bZIP19/23-independent Zn deficiency responses (Sinclair et al. 2018). In *Brachypodium distachyon*, analysis of *ZIP* genes expression and ionomics in the shoot of Zn-deficient plants upon resupply, also pointed at a root-to-shoot signal mediating the transcriptional response (Amini et al. 2021). The nature of these signals and long-distance signalling is not yet known in plants. In animals, it was proposed that “free” Zn ions can act as a signal, with several cell systems exhibiting dynamic changes in Zn concentrations that play a direct role in signalling pathways (Pratt et al. 2021).

## A tractable module for evolutionary and functional studies

### Conservation of the F-bZIP-regulated Zn deficiency response in land plants

A detailed phylogenetic analysis of F-bZIP homologs, in a set of 24 species representative of all major plant taxa, indicated evolutionary conservation of the Zn deficiency response across land plants (Castro et al. 2017). It showed that F-bZIPs are generally a small group of bZIP transcription factors and are present only in land plant species. F-bZIP members are present in non-vascular land plants, in mosses within the Bryophyte division, but were not found in the available genomes of green algae (Castro et al. 2017). This is in line with the proposal that F-bZIPs emerged in the first terrestrial plant lineage from founder genes of green plant ancestors (Corrêa et al. 2008). This may indicate a functional connection between the role of F-bZIPs in the Zn deficiency response and the colonization of the terrestrial environment (Castro et al. 2017). The evolution of higher organismal and morphological complexity associated with the transition to land includes new challenges for nutrient acquisition in terrestrial habitats (Wodniok et al. 2011). The availability of nutrients in the rhizosphere fluctuates depending on soil properties, environmental cues and temporal and spatial dynamics. It is thus conceivable that the F-bZIP-regulated Zn deficiency response played a role in the transition to land by equipping plants with a mechanism to cope with Zn deficiency within a range of variation in Zn availability from the soil.

The phylogenetic analysis across land plants indicated divergence of F-bZIP homologs during seed plant evolution into two groups (Castro et al. 2017), which was also supported by later phylogenetic analyses with enlarged datasets of Monocot and legume species (Lilay et al. 2020; Liao et al. 2022). These two groups, within F-bZIPs, were named Group 1 and Group 2. Interestingly, F-bZIPs from Group 1 are consistently present in all species, while Group 2 F-bZIPs are more prone to gene loss or expansion events, suggesting different selective pressures for each Group. The Arabidopsis bZIP19 and bZIP23, central regulators of the Zn deficiency response, belong to Group 1, while bZIP24, with no major role in this response (Lilay et al. 2019) and involved in salt stress regulation (Yang et al. 2009), belongs to Group 2 (Castro et al. 2017). Functional characterization of all annotated F-bZIP members from rice and *M. truncatula* indicated that rice OsbZIP48 and *M. truncatula* MtFbZIP1 belong to Group 1, and are the functional homologs of Arabidopsis AtbZIP19/23. The Group 2 F-bZIP OsbZIP49 from rice seems to be a truncated protein, and, together with the MtFbZIP2 from *M. truncatula*, they do not seem to be involved in the Zn deficiency response (Lilay et al. 2020; Liao et al. 2022). Finally, rice OsbZIP50, also from Group 2, does play a role in the Zn deficiency response, but its ectopic expression in Arabidopsis suggests an altered regulatory response at Zn sufficiency (Lilay et al. 2020). Thus, the combination of phylogenetic analysis and functional characterization of F-bZIPs supports the conservation of the Zn deficiency response associated with Group 1 members, while the Group 2 F-bZIPs show more variable evolutionary paths that can lead to non-, sub- or neo-functionalization (Castro et al. 2017). Nonetheless, all F-bZIP homologs analysed across land plants show a high level conservation of the ZSM (Assunção et al. 2013; Castro et al. 2017; Lilay et al. 2020; Liao et al. 2022), suggesting that possible neo-functionalization for Group 2 F-bZIPs might still retain a Zn-sensing function.

In addition to the phylogenetic analyses of F-bZIP homologs, analysis of the promoter region of Arabidopsis *ZIP4* homologs across land plants identified an enrichment in ZDREs and a conserved substitution in the consensus sequence (RTGT/ACGACAY) (Castro et al. 2017). The analysis of *ZIP* and *NAS* genes from barley, wheat, rice and *M. truncatula* revealed an association between Zn-deficiency induced expression and the presence of ZDREs in the promoter region (Evens et al. 2017; Nazri et al. 2017; Lilay et al. 2020; Liao et al. 2022). This further supports the conservation of the F-bZIP-regulated Zn deficiency response in land plants, which seems to comprise a conserved F-bZIP regulon.

### Sensor "on", a molecular switch for Zn biofortification?

Contrary to the native bZIP19/23 that act as sensors of the cellular Zn status, the bZIP19 ZSM variants become constitutively “on” (Fig. 2). The effect of such deregulation of the bZIP19/23 regulatory network on plant Zn content was analysed in plants grown at Zn sufficiency and, remarkably, it led to a significant increase in Zn concentration of shoots and seeds, the latter by 50%. This increase in Zn concentration was largely Zn-specific and with no apparent adverse effects on plant growth and development, under the conditions used with plants grown on normal soil (Lilay et al. 2021). The evolutionary conservation of the F-bZIP-regulated Zn deficiency response in land plants supports that the F-bZIP activity might be modulated, as a molecular switch, to improve Zn acquisition and biofortification also in crops. For example, through selection of naturally occurring ZSM variants in plant genetic resources or using genome precise edition tools. For this direction of research to produce useful biotechnological solutions for agriculture, detailed analyses will be needed to further clarify how ZSM variants affect plant Zn homeostasis, development and ionome composition and allocation patterns to edible organs in crops.

I finish by suggesting that the F-bZIP-regulated Zn deficiency response represents a very interesting tractable module for evolutionary and functional studies. The evolutionary conservation of the Cys/His-rich motif in F-bZIP homologs (Assunção et al. 2010) was the basis for the theoretical model proposing that Arabidopsis bZIP19 and bZIP23 can act as Zn sensors via a direct binding of Zn ions to the Cys/His-rich ZSM (Assunção et al. 2013). And recent findings reviewed here (Lilay et al. 2021), highlight a remarkable connection between evolutionary conservation and functional significance. The characteristics of this regulatory network, as per current evidence, such as its evolutionary conservation, generally low number of F-bZIP members, and a small set of target genes, make the F-bZIP-regulated Zn deficiency response a relatively simple system, and therefore a tractable module to combine evolutionary and functional studies across land plants. Comparative genomic approaches combined with protein functional analysis can, for example, shed light on the selective pressures and evolutionary fate of gene duplicates (e.g. mapped to F-bZIP Group 1 and Group 2), and advance knowledge on the properties and inter-relations of F-bZIP protein functional domains, such as the ZSM, the DNA binding region and its promoter ZDRE binding site, or the dimerization domains. It is also a tractable module to investigate the evolutionary significance of the F-bZIP-regulated Zn deficiency response in land colonization by plants, and importantly, to allow translational approaches for improving the Zn nutritional value of crop species.
